# Single Particle Fluorescence Burst Analysis of Epsin Induced Membrane Fission

**DOI:** 10.1371/journal.pone.0119563

**Published:** 2015-03-23

**Authors:** Arielle Brooks, Daniel Shoup, Lauren Kustigian, Jason Puchalla, Chavela M. Carr, Hays S. Rye

**Affiliations:** 1 Department of Biochemistry and Biophysics, Texas A&M University, College Station, Texas, United States of America; 2 Department of Physics, Princeton University, Princeton, New Jersey, United States of America; University of Edinburgh, UNITED KINGDOM

## Abstract

Vital cellular processes, from cell growth to synaptic transmission, rely on membrane-bounded carriers and vesicles to transport molecular cargo to and from specific intracellular compartments throughout the cell. Compartment-specific proteins are required for the final step, membrane fission, which releases the transport carrier from the intracellular compartment. The role of fission proteins, especially at intracellular locations and in non-neuronal cells, while informed by the dynamin-1 paradigm, remains to be resolved. In this study, we introduce a highly sensitive approach for the identification and analysis of membrane fission machinery, called burst analysis spectroscopy (BAS). BAS is a single particle, free-solution approach, well suited for quantitative measurements of membrane dynamics. Here, we use BAS to analyze membrane fission induced by the potent, fission-active ENTH domain of epsin. Using this method, we obtained temperature-dependent, time-resolved measurements of liposome size and concentration changes, even at sub-micromolar concentration of the epsin ENTH domain. We also uncovered, at 37°C, fission activity for the full-length epsin protein, supporting the argument that the membrane-fission activity observed with the ENTH domain represents a native function of the full-length epsin protein.

## Introduction

The parting and merging of lipid bilayers, as occur in vesicle budding (membrane fission) and in the delivery of vesicle contents to a target compartment (membrane fusion), are irreversible events. In order to impart specificity to the timing and integrity of each of these membrane remodeling events in the cell, proteins specialized to catalyze fission and fusion have evolved, as have regulatory factors, thus preventing indiscriminant events that would lead to intracellular disorganization[1–3].

While much progress has been made in the characterization of membrane fusion proteins [4–5], an understanding of the mechanism of membrane fission remains limited [3,6]. In part, this is due to the technical constraints of current methodologies. Bulk biochemical methods (such as sedimentation;[7–8]), tend to be inefficient, slow and provide only an estimate of the average observable activity of a complex system. While imaging methods can, in principle, circumvent this problem, to date these studies have either required intact cells, where detailed biochemical analysis of a system is not possible [9–12], are constrained by small event numbers [13], suffer from limited knowledge of population distributions and sampling bias, or are affected by surface perturbations of tethered objects [7,14]. Here we develop an alternative approach to the study of membrane fission, in which we apply a single particle fluorescence technique called Burst Analysis Spectroscopy (BAS; [15]). BAS permits analysis of the dynamics of complex particle distributions in free solution, including populations of liposomes undergoing fission. As a test of the utility of this approach for studying membrane fission, we have applied BAS to the study of changes in size and concentration of liposomes over time, when mixed with the simple, fission-potent, epsin N-terminal homology (ENTH) domain [7].

Epsin is a 94 kDa protein, identified in screens for binding partners of *α*-adaptin and Eps15, both clathrin coat associated proteins involved in clathrin-mediated endocytosis in neurons [16–17]. Epsin is generally believed to function in cargo selection and bud site nucleation, through direct interactions with Eps15, the clathrin adaptor protein, AP-2, endocytic cargo and with clathrin, itself (reviewed in ref.[18]). At the amino terminus of epsin is the highly conserved, ~140 amino acid ENTH domain shared with other endocytic proteins, including AP180/CALM [19]. This domain contains an N-terminal amphiphathic helix (the H_0_ helix), which is known to insert into the outer-leaflet of membranes in a PtdIns(4,5)P_2_-dependent fashion [20]. Membrane insertion of the H_0_ helix is thought to facilitate membrane curvature and tubulation, prior to fission.

Recently, it was suggested that insertion of the ENTH H_0_ helix into a lipid bilayer could directly facilitate fission [7]. This work reported potent fission activity when liposomes were mixed with the isolated ENTH domain, though full-length epsin did not appear to possess fission activity. Recently, the conclusions derived from those results have been called into question: the observed ENTH-domain activity was suggested to be an artifact of a non-native protein domain at high concentration interacting non-specifically with liposomes to generate small vesicles and/or micelles [8].

Here we have reexamined liposome membrane fission mediated by the ENTH-domain and full-length epsin protein with BAS. We find that the rate of membrane fission by the ENTH domain is sensitive to both temperature and protein concentration, and that fission activity can be observed at a sub-micromolar protein concentration, comparable to studies of dynamin-2 [8]. By exploiting the inherent sensitivity of BAS, we also observed measurable membrane fission activity for full-length epsin, albeit attenuated when compared to the ENTH domain. These observations not only support the argument that membrane fission is a function of full-length epsin, but also demonstrate that BAS offers a flexible and highly sensitive approach to the study of membrane dynamics.

## Experimental Procedures

*Abbreviations—*BAS, Burst Analysis Spectroscopy; TIRF, total internal reflection; ENTH, epsin N-terminal homology; IPTG, isopropyl thiogalactose; DTT, dithiothreitol; FCS, fluorescence correlation spectroscopy; PtdEth, phosphatidyl ethanolamine; PtdChl, phosphatidyl choline; PtdIns, phosphatidyl inositol; P, phosphate.

### Protein expression and purification

The coding sequence of the epsin ENTH domain (residues 1–164) from *Rattus norvegicus* was obtained from Addgene and was sub-cloned into the pPROEX HTb vector [21] for expression in *E*. *coli* BL21. In brief, clarified lysates were run on a Ni-NTA column equilibrated with column buffer (20 mM Tris pH 8.0, 500 mM NaCl, 20 mM imidazole, 5 mM β-mercaptoethanol) and eluted with a step gradient of the same buffer, plus 500 mM imidazole. ENTH-domain containing fractions were pooled and dialyzed against column buffer with 0.4 μM His_6_-TEV protease. His_6_-TEV and the His_6_ tag were separated from the untagged protein using the nickel affinity column. Untagged ENTH domain from the flow-through was further purified by ion exchange chromatography on a Source S column equilibrated in Source S Buffer A (20 mM Tris pH 7.4, 2 mM DTT) and eluted with a linear gradient of Source S Buffer B (20 mM Tris pH 7.4, 2 M NaCl, 2 mM DTT). Purified ENTH was stored at -80°C, in 20 mM Tris pH 7.4, 150 mM KCl, 2 mM DTT. The coding sequence of full-length rat epsin was obtained from Addgene (pCDNA3.1-Epsin1; plasmid 22225) and was sub-cloned into the pEX-N-His_6_-GST vector (Origene) for expression in *E*. *coli* BL21[DE3]. Purification of full length epsin followed the same affinity chromatography and proteolytic cleavage protocol used for the ENTH domain, followed by further purification by ion exchange with a high-resolution Mono Q column equilibrated and washed with Buffer A (20 mM Tris pH 7.4, 2 mM DTT) and eluted on a linear gradient with Buffer B (20 mM Tris pH 7.4, 2 mM DTT, 2 M NaCl).

### Liposome preparation

Liposomes were prepared as previously described, with minor modifications [7]. Briefly, brain lipid Folch extracts from Avanti (cat. 131101P) and Sigma (cat. B-1502) were mixed 1:1, with 5% PtdIns(4,5)P_2_ (Avanti, cat. 840046C) and 0.03% acyl-chain, Ω-carbon labeled TopFluor-PtdEth (Avanti, cat. 810282C). Lipids were dried under a stream of dry argon, vacuum desiccated to remove residual solvents, re-suspended, with freezing and thawing, to 1 mg/ml in liposome buffer (20 mM HEPES pH 7.4, 150 mM NaCl) and then extruded through polycarbonate filters with the indicated diameters with 11 passes in a mini extruder (Avanti), followed by 10 passes through a high-pressure manifold extruder (Northern Lipids), and used within 6 hr. Liposomes used at later times no longer respond to addition of ENTH domain or epsin, presumably due to loss of liposome binding upon PtdIns(4,5)P_2_ hydrolysis.

### Liposome fission assay by BAS

Liposomes diluted to 0.01 mg/mL in liposome buffer were mixed with ENTH domain, or full-length epsin, at the concentrations indicated, and 10 μL of each sample was spotted onto a BSA-blocked glass coverslip held in a custom cassette. The coverslip cassette was clamped to a high-precision, computer controlled, 2-axis translation stage connected to a customized microscope system, and data were collected as previously described [15,22]. Efficient fission of large (~ 200 nm) liposomes into small (20–30 nm) liposomes should result in a large (100 to 200-fold) increase in object concentration, read out as fluorescent bursts with amplitudes proportional to individual object sizes. From a starting sample of 50–100 pM large liposomes, this increase in object number will violate the single-particle concentration limit (< 500 pM) required for BAS. Additionally, due to limited knowledge of the instrument point spread function, an individual BAS measurement can only quantitatively probe an approximately 100-fold range in object intensity for a single, uniformly labeled species [15]. The fission of large liposomes into much smaller ones leads to a highly inverted population dominated by smaller particles. In this case, the resolving power of BAS deteriorates for the low intensity events, due to the high species concentrations that no longer permit single particle detection. Therefore, to accurately examine liposome populations produced during fission, we developed an enhanced measurement protocol that permits BAS histograms to be constructed over an arbitrarily large range of object sizes. In brief, standard BAS data are collected on a series of systematic dilutions of each reaction sample, followed by analytical reconstruction of the overall population distribution through simultaneous fitting of the object cumulative distributions across the dilution series. Our standard BAS analysis fitting routines [15] have been modified to accommodate this expanded data analysis strategy. The fitting and programmatic details will be published elsewhere.

### Heat maps

Plots representing the spread of liposome products as a function of time or concentration are shown in some cases as “heat maps”: a stack of rows, one experiment per row, with increasing brightness corresponding to an increased fractional intensity of each bin (where a bin represents a group of burst events of a given size). To convert the number of burst events in each bin to fractional intensity, we normalized the object intensities as follows:

$$\frac{I_{i}C_{i}}{\sum_{i=1}^{n}I_{n}C_{n}}$$ (1)

where I_i_ is the intensity of each bin, C_i_ is the concentration of objects in each bin, and the denominator represents the total fluorescence of all bins (the sum of intensity in a row) for a given sample.

## Results

### BAS is sensitive to changes in liposome size and concentration

**Fig 1:**
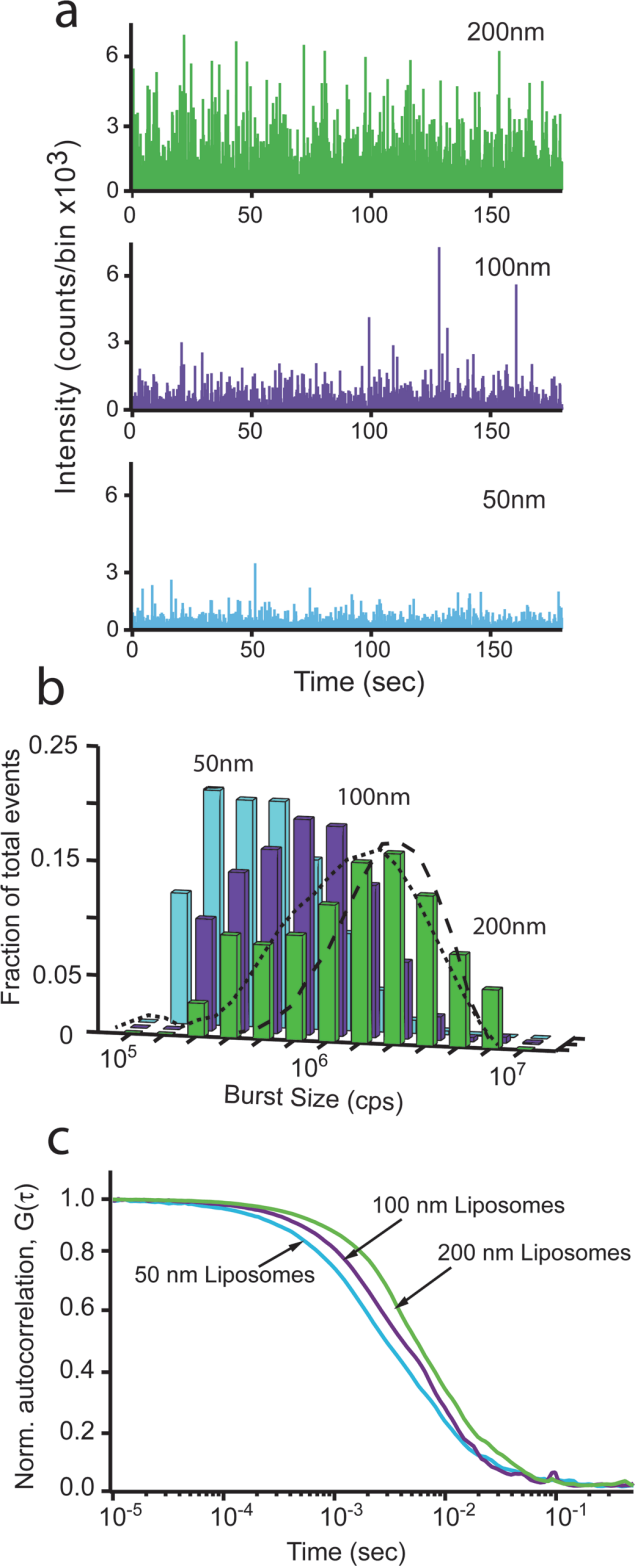
BAS assay distinguishes liposomes of different sizes. The size distribution of 200 nm, 100 nm and 50 nm fluorescent liposomes was examined by FCS and BAS. (a) Fluorescent burst data of TopFluor-labeled Folch liposomes extruded to 200 nm (*green*), 100 nm (*purple*) and 50 nm (*cyan*). (b) BAS histograms generated from the burst data in (a). Fraction of total events is the concentration of each bin divided by the total concentration, for each sample. Dashed lines show theoretical diameter distributions (35% CV, *dash*; 50% CV, *dot*) derived from Monte Carlo simulated intensity data in which fluorescence brightness was set proportional to particle surface area. The resulting simulated intensity distributions were analyzed with BAS analysis code. (c) FCS profiles of 200 nm, 100 nm, and 50 nm liposomes. The data shown is representative of two experimental replicates.

In order to calibrate our BAS measurements of membrane fission, we first examined a series of liposome standards created with different diameters. Liposomes were extruded to 200 nm, 100 nm, and 50 nm and then examined by BAS. Single-particle burst data for these samples display the expected dependence of burst size on liposome size (Fig. 1A). The fluorescence intensity of these membrane labeled liposomes is expected to be proportional to their surface area; thus, the mean intensity of the 200 nm versus 100 nm, as well as the 100 nm versus the 50 nm liposomes should differ by ~ 4-fold and the 200 nm and 50 nm are expected to differ by ~ 16-fold. As shown in Fig. 1B, the BAS histograms of each liposome sample display distributions with mean intensity differences consistent with the expected values. Additionally, the dispersion in liposome sizes measured by BAS is consistent with the expected variation for liposomes created by extrusion, specified as ± 25% CV (coefficient of variation; Northern Lipids specifications). As shown in Fig. 1B, the observed size variation in liposomes appears to be between 35–50% CV, based on Monte Carlo simulations of particle distributions in which the particle brightness is assumed to be proportional to surface area. While the observed liposome variation is somewhat larger than expected, several factors likely broaden the observed intensity distribution, including a small fraction of multi-lamellar objects [23] and the discreet distribution of dye molecules between objects of the same absolute size. As a complementary measurement, we examined each liposome sample by FCS (Fig. 1C). FCS provides information on hydrodynamic radius based on measurement of the average diffusion time of fluorescent objects as they diffuse in and out of the excitation beam. However, diffusion time increases linearly with particle radius and so is not as sensitive a measure of liposome size as fluorescence intensity, which increases with the square of the radius. Additionally, FCS is dependent on particle surface hydrophobicity so that the hydrodynamic radius can be converted to an effective size only with knowledge of this surface-solvent interaction. Therefore, we use FCS here primarily as an indicator of a difference in average population hydrodynamic radius. Consistent with the BAS measurements, the mean diffusion time for each liposome sample decreases as the extrusion filter pore diameter decreases (Fig. 1C).

### Membrane fission activity of the Epsin ENTH domain

**Fig 2:**
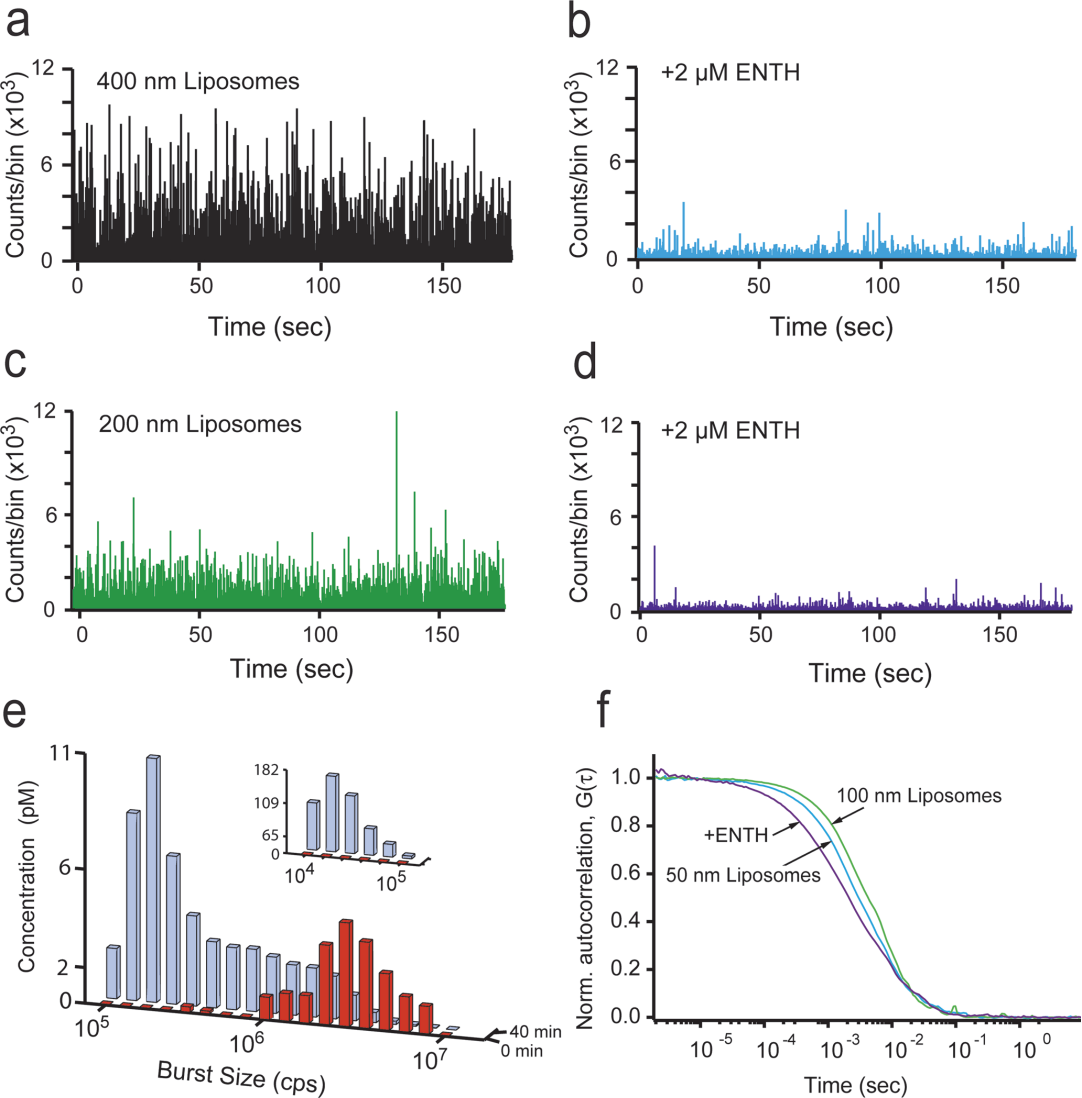
BAS analysis of liposomes vesiculated by the ENTH domain of epsin. Fluorescent burst data for 400 nm-diameter, TopFluor-labeled, (5%) PtdInsP(4,5)P_2_ Folch liposomes incubated at 37°C for 40 min before (a) and after addition of 2 μM ENTH (b). Fluorescent burst data for 200 nm-diameter liposomes incubated at 37°C for 40 min before (c) and after addition of 2 μM ENTH (d). (e) BAS histograms generated from starting 200 nm liposomes before (*red*) and after addition of ENTH (*blue*; insets indicate resolution of small particles in a 10-fold dilution of the same reaction). (f) FCS profiles of liposomes extruded to 100 nm (*green*), 50 nm (*cyan*) and the end products (*purple*) of the fission reaction of 200 nm liposomes from (d). The data shown is representative of three experimental replicates.

We next examined the ability of BAS to detect products of ENTH domain-mediated fission. Samples of large liposomes (either 400 nm or 200 nm) were mixed with purified ENTH domain and then examined by BAS after 40 min incubation at 37°C. We anticipated fission to be detectable as a shift from a small number of large fluorescence bursts to a larger number (high concentration) of much smaller bursts. As shown in Fig. 2 A-D, the expected changes are observed upon addition of the ENTH domain to either 400 nm, or 200 nm liposomes. Importantly, the total fluorescence intensity of the sample before and after ENTH addition changed by no more than 10–20% (S1 Fig.), confirming that the disappearance of the large bursts was not caused by a loss of the starting liposomes, but rather by their conversion into a high concentration of smaller objects.

The extent of fission of the 200 nm liposomes was quantified by BAS analysis of the raw burst data. The resulting BAS histograms display a dramatic shift from a low concentration of large liposomes to an increased concentration of small ones (Fig. 2E). The smallest products of the fission process (Fig. 2E, inset) increase by over 100-fold relative to the staring concentration of 200 nm liposomes, consistent with the number of small liposomes expected from efficient fission of the starting 200 nm liposomes into ~ 20 nm products. A similar scaling argument predicts that the mean burst size of a 20 nm product liposome should be ~ 100-fold smaller than the mean burst size of a starting 200 nm liposome, assuming that segregation of the fluorescent label is not biased by the process of fission. As shown in Fig. 2E, the relative difference in mean burst size for the staring and product liposomes is consistent with the product liposomes being ~ 20 nm in size. The product liposome distribution is most consistent with an approximately 30% CV (20 nm ± 6 nm), based on comparison of the intensity variation in the smallest product liposomes with simulated particle populations created at different size variations (10–50% CV; see Fig. 1B for an example). Examination of samples by FCS is also consistent with efficient membrane fission. Liposomes mixed with the ENTH domain show a dramatic shift in average diffusion time to values substantially less than that observed for 50 nm liposomes (Fig. 2F). However, similar experiments in which the ENTH domain was replaced with a non-specific protein (bovine serum albumin; BSA) displayed no detectable fission activity (S2 Fig.), indicating that the observed shift in liposome size requires a potent, fission-active protein. Taken together, these observations are consistent with the generation of ~ 20 nm vesicles by the ENTH domain, as previously observed by electron microscopy [7].

### The ENTH domain acts on the timescale of minutes

**Fig 3:**
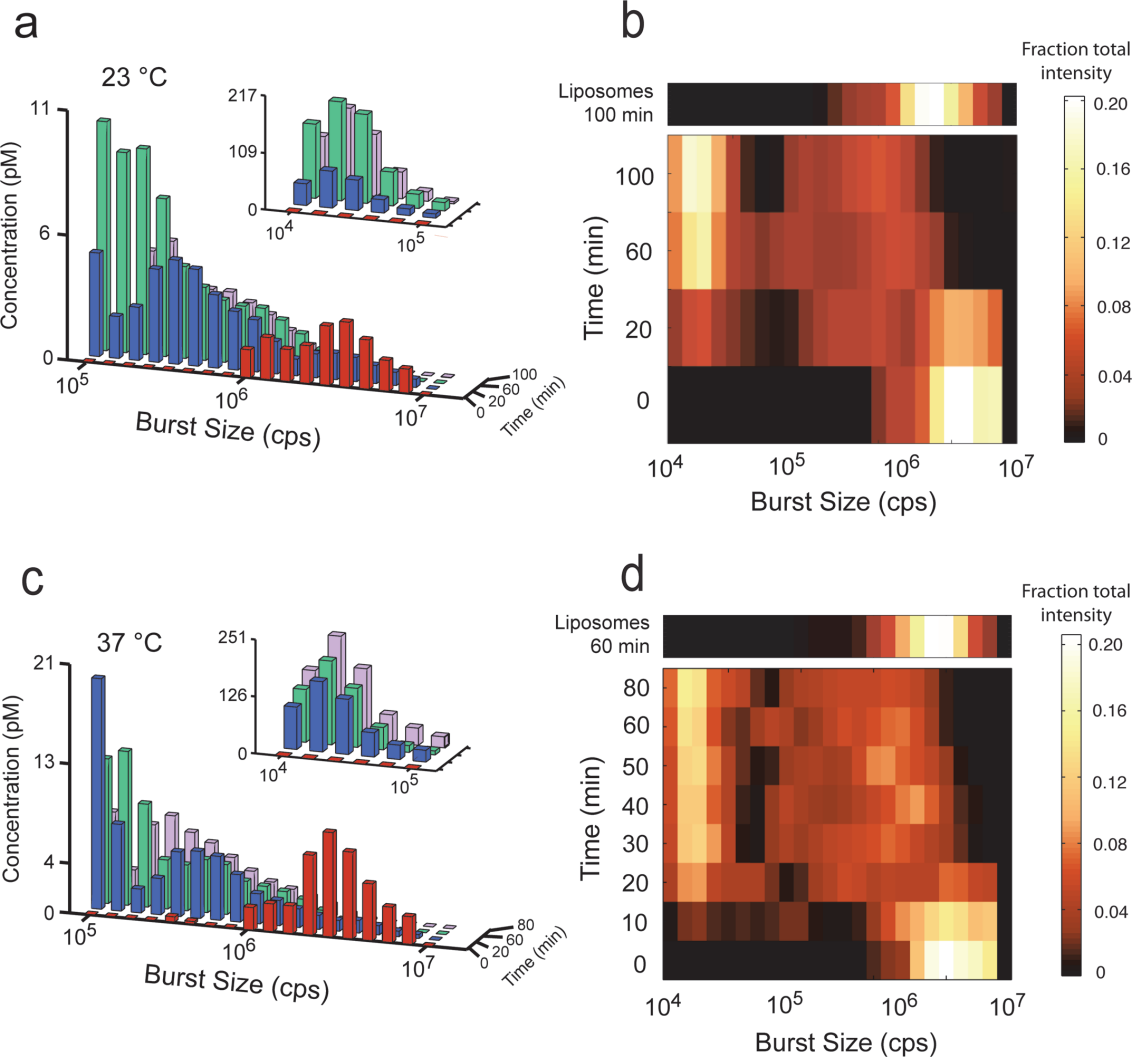
Kinetics of liposome fission are temperature dependent. (a) Histograms of BAS analyzed 200 nm-diameter, TopFluor-labeled, (5%) PtdInsP(4,5)P_2_ Folch liposomes (*red*) and products of ENTH incubation at 23°C for 20 (*blue*), 60 (*green*), and 100 min (*purple*) after addition of 2 μM ENTH. At each time point, an aliquot was removed and placed on ice, and measurements were started within 1–2 min. Inset indicates resolution of small particles in a 10-fold dilution of each reaction. (b) Heat map representation of the fractional intensity for each reaction shown in (a). (c) BAS histograms generated from starting liposomes (*red*) and products of ENTH incubation at 37°C for 20 (*blue*), 60 (*green*), and 80 min (*purple*) after addition of 2 μM ENTH. (d) Heat-map representation of the fractional intensity for each reaction shown in (c). Additional time points are shown, for increased resolution. The effect of incubating liposomes in the absence of the ENTH domain for 60 min at 37°C or 100 min at 23°C is shown as an additional row, above the respective heat maps. The data shown for the experiments conducted at 23°C is representative of four experimental replicates. The data shown for experiments conducted at 37°C is representative of three experimental replicates.

The sensitivity of BAS permits changes in the liposome population as a function of time to be mapped with far greater accuracy than achieved previously. After 20 min at 23°C, a significant shift in the vesicle population size distribution is observed; however, some large vesicles remain (Fig. 3A). By 60 min, the largest vesicles are observed rarely, and the disappearance of large vesicles is concurrent with the appearance of smaller ones, over the 100 min time course. The fission activity of the ENTH domain is enhanced at 37°C (Fig. 3C), with the largest vesicles observed rarely at 20 min. By 80 min, at 37°C, the reaction appears to be complete, matching the 100 min time point at 23°C. In order to more fully illustrate the changes in liposome populations as a function of time, we normalized the fractional intensity of each BAS intensity bin and re-plotted the data as a heat map (see methods; Fig. 3B, 3D). While the large, starting liposomes at time zero form a bright peak on the right end of the plot, at later time points, the fractional fluorescence is distributed between small and medium products, eventually populating a bright peak of small liposomes at the top (left), plus a lower concentration of broadly distributed, medium liposome sizes. Whether these intermediate-sized liposomes are static end products or the result of additional liposome dynamics is unknown, but they persist regardless of protein concentration or time. Interestingly, the rate at which the largest liposome population disappears does not appear to be detectably different at 23°C and 37°C. This observation is likely due to the small fractional change in intensity that occurs when 20 nm liposomes split from the much larger 200 nm liposome objects. The resulting 1–2% change in fluorescence intensity is not detectable in this assay, given the difference in the rate of fission at the two temperatures. However, the smallest products reach their maximum concentration at ~ 30 min at 37°C and ~ 60 min at 23°C, indicating a ~ 2-fold increased rate at 37°C.

### Fission activity of the ENTH domain is dose-dependent

**Fig 4:**
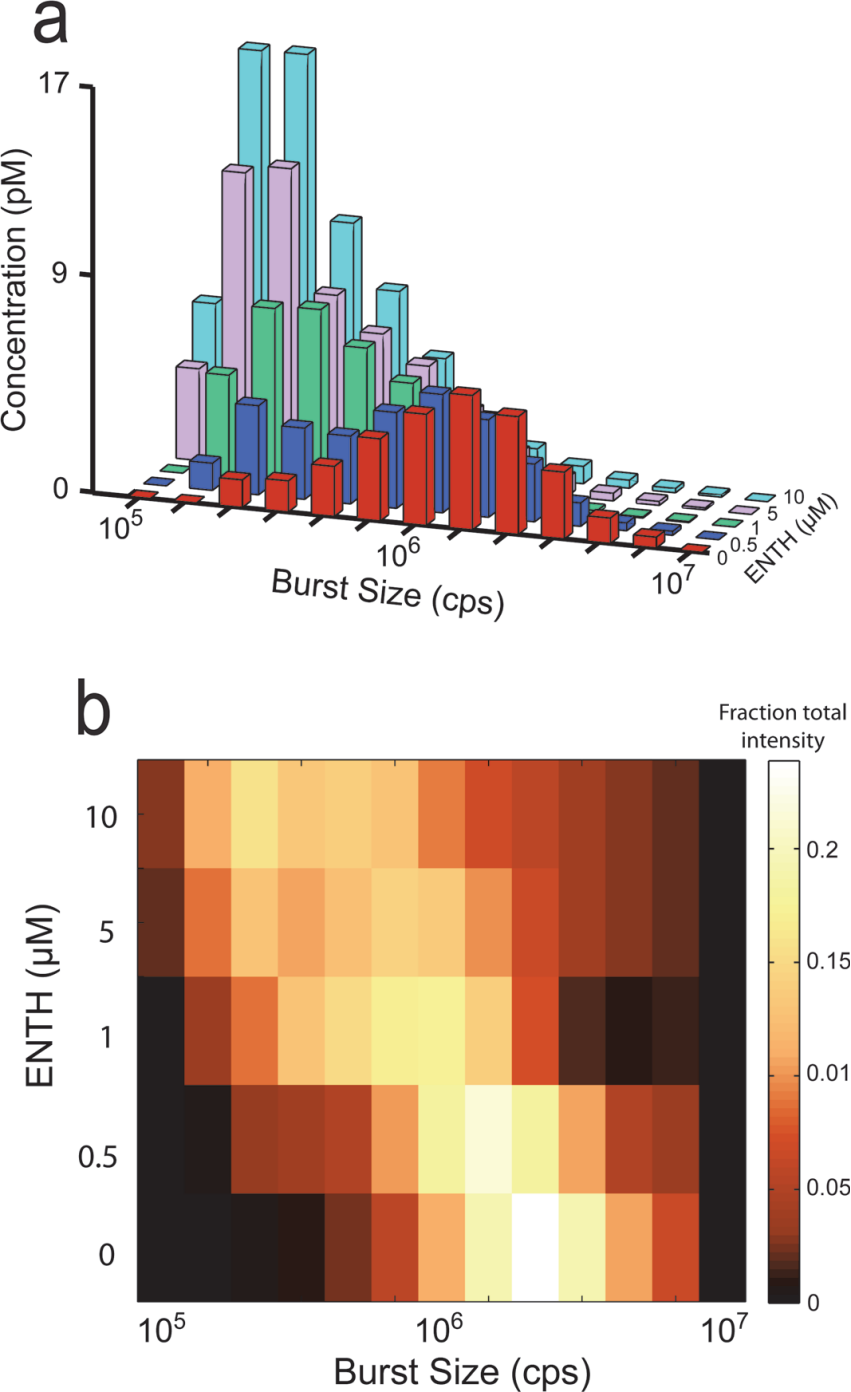
Dose dependence of ENTH-mediated vesiculation. (a) BAS histograms of 200 nm-diameter, TopFluor-labeled, (5%) PtdInsP(4,5)P_2_ Folch liposomes before (*red*) and after incubation at 37°C for 20 min with 500 nM (*blue*), 1 μM (*green*), 5μM (*purple*), and 10 μM (*cyan*) ENTH. (b) Heat-map representation of the fractional intensity for each reaction shown in (a). The data shown is representative of three experimental replicates.

Next, we looked for a dose-dependent change in fission activity of the ENTH domain. In order to maximize the sensitivity of observable changes in the liposome size-distribution, we chose an early time point in the fission reaction (20 min at 37°C) and focused on the disappearance of large liposomes and appearance of intermediate-sized products (without the dilutions required to resolve small products). Using this approach, we were able to measure fission activity at concentrations as low as 500 nM ENTH domain (Fig. 4A, 4B). At this early time point, the fission activity increases as a function of protein concentration, up to 10 μM (Fig. 4A, 4B).

### Fission activity of full-length epsin

**Fig 5:**
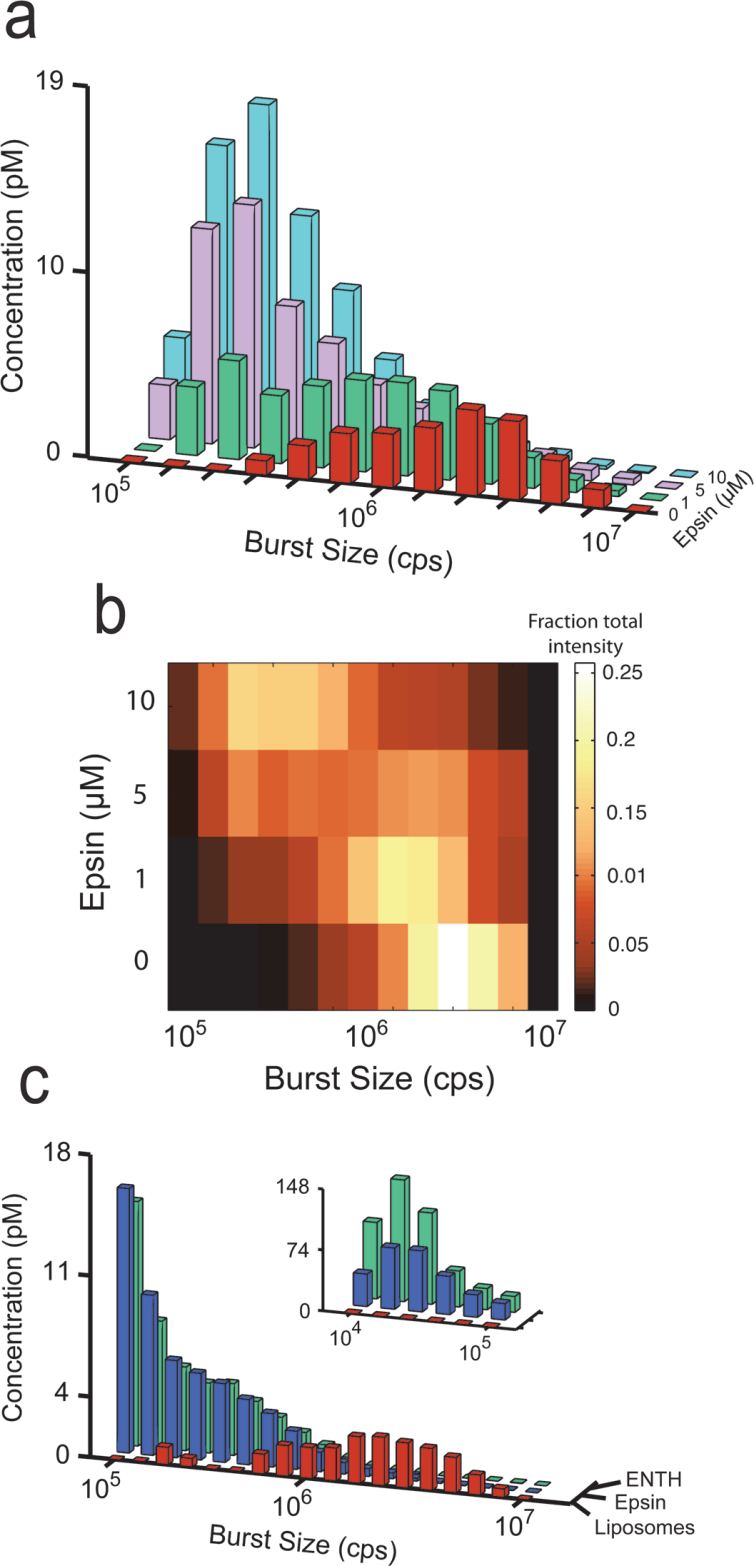
Full-length epsin has vesicle fission activity. (a) BAS histograms of 200 nm-diameter, TopFluor-labeled, (5%) PtdInsP(4,5)P_2_ Folch liposomes before (*red*) and after incubation at 37°C for 40 min with 1 μM (*green*), 5 μM (*purple*), and 10 μM (*blue*) full-length epsin. (b) Heat-map representation of the fractional intensity for each reaction shown in (a). (c) Comparison of ENTH and epsin activity. BAS histograms of starting liposomes (*red*), and liposomes incubated at 37°C for 90 min with 2 μM ENTH (*blue*) or full-length epsin (*green*). The data shown is representative of three experimental replicates.

While the results of these and previous studies [7] indicate potent membrane fission activity for the epsin ENTH domain, it remained possible that the activity we observed is an artifact of the truncation and not a function of the full-length epsin protein. We reasoned that if epsin has latent membrane fission activity, then we might uncover that activity using the high sensitivity of BAS and conditions that maximize fission activity for the ENTH domain. Using this approach, we observed dose-dependent liposome fission activity at epsin concentrations as low as 1 μM, albeit with significantly slower rates than observed for the ENTH domain (Fig. 5A, 5B). To compensate for the slower rates, the dose-dependence of epsin activity was measured at a 40 min time point and compared to the earlier, 20 min time point, used for the ENTH domain (Fig. 4). Despite the kinetic differences, the distribution of liposome products is remarkably similar and converges to the same size end products, after a 90 min incubation. These results suggest that both the ENTH domain and the full-length epsin protein employ the same membrane-fission mechanism (Fig. 5C).

## Discussion

Using BAS, we observed time-resolved liposome membrane fission in free solution, induced by the potent epsin ENTH domain. These results agree with those of a previous study, which showed, using living cells and an *in vitro* sedimentation assay, that epsin, in particular the ENTH domain, is necessary and sufficient for endocytic vesicle membrane fission [7]. Recently, concerns were raised regarding the physiological significance of the fission activity observed in that study, specifically citing the small size of the starting liposomes (200 nm diameter), the high protein concentration (10 μM), and the likelihood that many of the products, rather than vesicular in nature, were micellar [8]. The high sensitivity of BAS allowed us to address these concerns: (i) fission activity was observed at sub-micromolar protein concentration, (ii) fission activity does not depend on the curvature of starting liposomes, as those of 400 nm diameter worked as well as smaller ones and (iii) the products are consistent with 20 nm vesicles and not micelles, as observed previously [7].

In addition, the high sensitivity of BAS allowed us to uncover attenuated membrane fission activity in experiments with the full-length epsin protein. Attenuation may suggest an inhibited conformation for full-length epsin, as has been suggested for syndapin, another protein involved in formation of vesicles at the recycling endosome [24]. Intermolecular interactions have also been observed to cause auto-inhibition, in the case of endophilin A1, a curvature-inducing endocytic protein that also contains an N-terminal amphipathic helix [25].

Although epsin is required for clathrin-mediated endocytosis from early to late stages of endocytic vesicle formation [12], it has been classified as an adaptor protein. Like the well-characterized adaptins, epsin was shown to act at an early step, recruiting other adaptins and cargo [18], in addition to binding to the clathrin coat. However, unlike the classic adaptins, little epsin is found in clathrin-coated vesicles [17,26], raising questions regarding its role solely as an adaptor protein. Furthermore, epsin can rescue a block in the release of endocytic vesicles in dynamin-depleted cells [7], arguing for a late role in vesicle fission. These results, in addition to the liposome fission activity of the epsin ENTH domain, led those authors to conclude that epsin is also required for fission of clathrin-coated endocytic vesicles. Furthermore, an analogy was made to the early and late requirement for the amphipathic-helix containing proteins, Arf1p and Sar1p, in the formation of COPI coated vesicles at the Golgi apparatus and COPII coated vesicles at the endoplasmic reticulum, respectively [27,28].

BAS has the ability to resolve membrane fission reactions over a concentration range that is more physiologically significant and on a sub-second timescale. Yet, the fastest fission reactions we observed, at high protein concentration and 37°C, proceeded on the minute, to tens of minutes timescale. This suggests that other factors are required to increase the fission activity to physiologically significant rates, on the order of seconds, to tens of seconds [12]. Notably, the results of recent studies indicated a reciprocal requirement for the amphipathic-helix containing amphiphysin and partner protein, dynamin, in order to stimulate membrane fission [8,29]. Our results using BAS reopen the question of how membrane fission is induced, not only in endocytosis, but also, how transport carriers and vesicles are released at other locations in the cell, where dynamin does not appear to play a role. Our findings also raise others questions: how is epsin regulated, and what stimulates epsin-induced membrane fission? Moreover, our findings indicate that BAS offers a highly sensitive approach to follow single particle dynamics of a membrane fission reactions in free-solution, for identification of membrane fission agents and characterization of the mechanism of membrane fission regulation.

## Supporting Information

S1 Fig Liposome fission by ENTH is not accompanied by loss of fluorescent material. The total fluorescent signal for each time point during ENTH-mediated fission at 37°C (Fig. 3C) is shown. In each case, the integrated signal was normalized to the maximal total signal observed during the experiment. The data shown is representative of three experimental replicates.
(TIF)

S2 Fig Liposome size distribution is not altered by a non-fission active protein. A BAS histogram of 200 nm-diameter, TopFluor-labeled, (5%) PtdInsP(4,5)P_2_ Folch liposomes (*light blue*) remains relatively unchanged, following a 60 min incubation at 37°C in the presence of 10 μM BSA (*red*). The data shown is representative of duplicate experiments.
(TIF)
