# Supplementary figures and images for: Single Particle Fluorescence Burst Analysis of Epsin Induced Membrane Fission
Source: PLoS One. 2015 Mar 23;10(3):e0119563. doi: 10.1371/journal.pone.0119563 (PMC4370887; doi:10.1371/journal.pone.0119563)

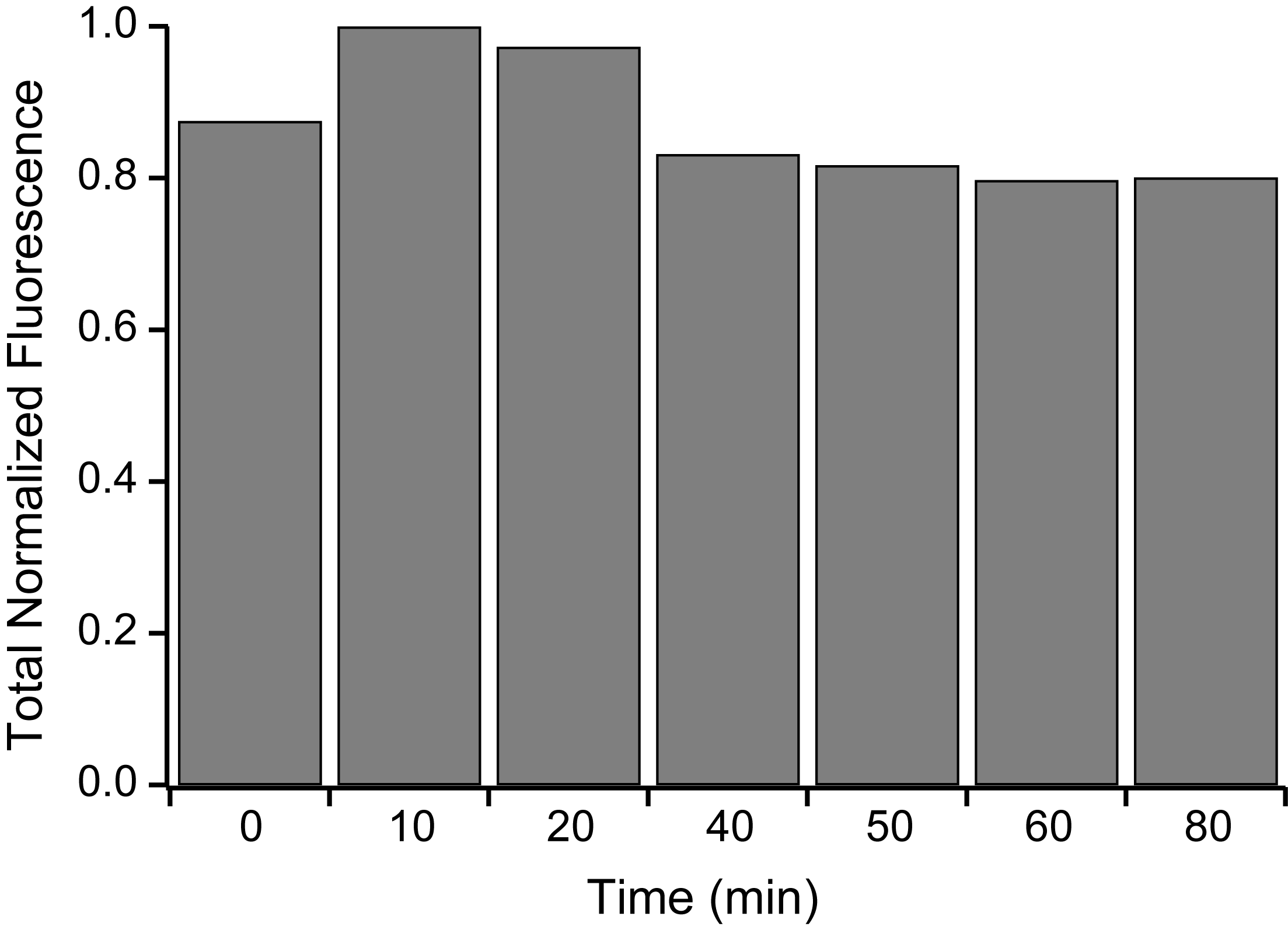

Supplement: S1 Fig — The total fluorescent signal for each time point during ENTH-mediated fission at 37°C (Fig. 3C) is shown. In each case, the integrated signal was normalized to the maximal total signal observed during the experiment. The data shown is representative of three experimental replicates. (TIF) [file pone.0119563.s001.tif]

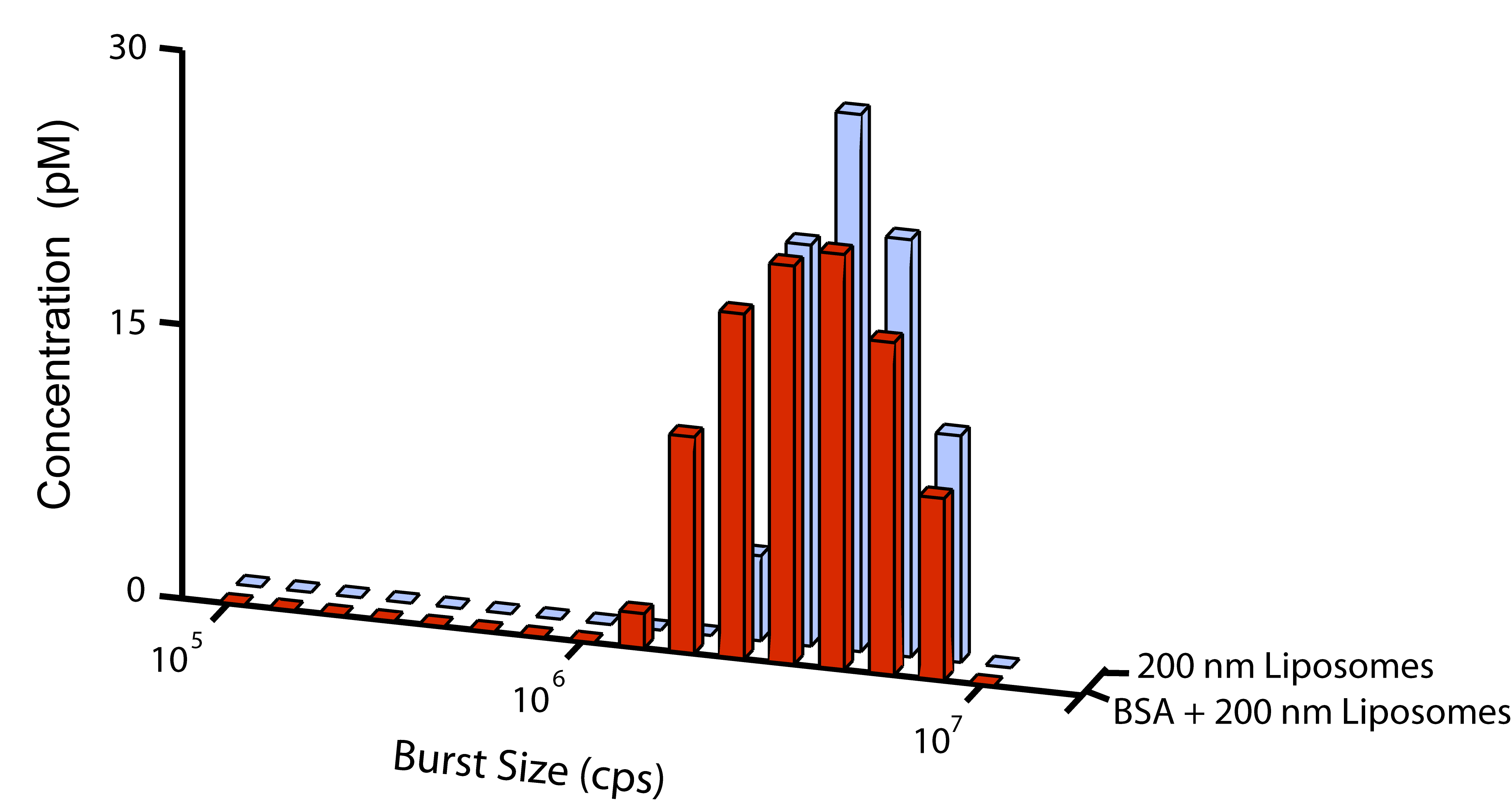

Supplement: S2 Fig — A BAS histogram of 200 nm-diameter, TopFluor-labeled, (5%) PtdInsP(4,5)P2 Folch liposomes (light blue) remains relatively unchanged, following a 60 min incubation at 37°C in the presence of 10 μM BSA (red). The data shown is representative of duplicate experiments. (TIF) [file pone.0119563.s002.tif]
